# A Visualized Dynamic Prediction Model for Lymphatic Metastasis in Ewing's Sarcoma for Smart Medical Services

**DOI:** 10.3389/fpubh.2022.877736

**Published:** 2022-05-04

**Authors:** Wenle Li, Chan Xu, Zhaohui Hu, Shengtao Dong, Haosheng Wang, Qiang Liu, Zhi-Ri Tang, Wanying Li, Bing Wang, Zhi Lei, Chengliang Yin

**Affiliations:** ^1^Department of Orthopedics, Xianyang Central Hospital, Xianyang, China; ^2^Clinical Medical Research Center, Xianyang Central Hospital, Xianyang, China; ^3^Department of Spinal Surgery, Liuzhou People's Hospital, Liuzhou, China; ^4^Department of Spine Surgery, Second Affiliated Hospital of Dalian Medical University, Dalian, China; ^5^Department of Orthopaedics, The Second Hospital of Jilin University, Changchun, China; ^6^School of Physics and Technology, Wuhan University, Wuhan, China; ^7^Chronic Disease Division, Luzhou Center for Disease Control and Prevention, Luzhou, China; ^8^Faculty of Medicine, Macau University of Science and Technology, Taipa, Macau SAR, China

**Keywords:** Ewing's sarcoma (ES), SEER database, multicenter data, nomogram, web calculator

## Abstract

**Background:**

This study aims to predict the lymphatic metastasis in Ewing's sarcoma (ES) patients by nomogram. The risk of lymphatic metastasis in patients with ES was predicted by the built model, which provided guidance for the clinical diagnosis and treatment planning.

**Methods:**

A total of 929 patients diagnosed with ES were enrolled from the year of 2010 to 2016 in the Surveillance, Epidemiology, and End Results (SEER) database. The nomogram was established to determine predictive factors of lymphatic metastasis according to univariate and multivariate logistic regression analysis. The validation of the model performed using multicenter data (*n* = 51). Receiver operating characteristics (ROC) curves and calibration plots were used to evaluate the prediction accuracy of the nomogram. Decision curve analysis (DCA) was implemented to illustrate the practicability of the nomogram clinical application. Based on the nomogram, we established a web calculator to visualize the risk of lymphatic metastases. We further plotted Kaplan-Meier overall survival (OS) curves to compare the survival time of patients with and without lymphatic metastasis.

**Results:**

In this study, the nomogram was established based on six significant factors (survival time, race, T stage, M stage, surgery, and lung metastasis), which were identified for lymphatic metastasis in ES patients. The model showed significant diagnostic accuracy with the value of the area under the curve (AUC) was 0.743 (95%CI: 0.714–0.771) for SEER internal validation and 0.763 (95%CI: 0.623–0.871) for multicenter data external validation. The calibration plot and DCA indicated that the model had vital clinical application value.

**Conclusion:**

In this study, we constructed and developed a nomogram with risk factors to predict lymphatic metastasis in ES patients and validated accuracy of itself. We found T stage (Tx OR = 2.540, 95%CI = 1.433–4.503, *P* < 0.01), M stage (M1, OR = 2.061, 95%CI = 1.189–3.573, *P* < 0.05) and survival time (OR = 0.982, 95%CI = 0.972–0.992, *P* < 0.001) were important independent factors for lymphatic metastasis in ES patients. Furthermore, survival time in patients with lymphatic metastasis or unclear situation (*P* < 0.0001) was significantly lower. It can help clinicians make better decisions to provide more accurate prognosis and treatment for ES patients.

## Introduction

Ewing's sarcoma (ES) is the secondly common malignant bone and soft tissue tumor in children and adolescents, with an incidence of 1 in 1.5 million. It is a small round cell malignant tumor originating from bone marrow mesenchymal stem cells. Eighty-five to ninety percent of ES patients have typical chromosomal translocations [*t*_(11;22)__(q24;q12)_] and the expression of EWS-FLI1. It is characterized by young onset age, non-specific clinical features, strong invasion ability and poor clinical prognosis (1–5). ES often occurs in the epiphysis of the extremities, mainly presenting as swelling with palpable mass and mild pain, accompanied by obvious night pain and pathological fracture, which seriously affects the quality of life of patients. Early diagnosis lacks specificity with a high misdiagnosis rate (5–8).

Patients with ES have poor long-term prognosis in spite of the variety of treatments and the progress made in surgery, radiotherapy and chemotherapy (1, 9, 10). Up to 80% of patients with ES have distant metastasis at initial diagnosis, whose overall 5-year survival rate is <30%. Although the application of surgery and multi-agent neoadjuvant chemotherapy has resulted in a significant improvement in the prognosis of patients with ES, the survival prognosis of patients with recurrence and metastasis remains poor (11, 12).

Few previous studies have involved combining multiple independent factors to predict lymphatic metastasis in patients with ES (13, 14). Therefore, accurate prediction of the probability of lymphatic metastasis has high application value. As a prediction model based on independent factors of clinical prognosis, nomogram has good stability and applicability (15, 16). Therefore, it has been widely used to evaluate tumor prognosis and metastasis (17–21). Likewise, it can be used to predict and evaluate the prognosis of patients with ES. Besides, it can help improve early detection, disease surveillance, overall patient survival rate and their life quality.

In this study, the patients' data was extracted from the SEER database in order to establish a nomogram. Furthermore, we validated it externally via multi-center clinical data of patients. To provide patients with more accurate medical services, we also designed a web calculator that could help clinicians quickly and accurately assess the risk of lymphatic metastasis in various ES patients.

## Materials and Methods

### Retrieval From Public Databases

The Surveillance, Epidemiology, and End Results (SEER) database was used for data collection and analysis. The database is the most detailed and authoritative one which provide evidence of evidence-based medicine, covering more than 30% of the total population in the United States from 1973 to 2016. The patients in this study were selected from the SEER database for diagnosis of ES from 2010 to 2016.

### The Collection of Clinical Data

Patients diagnosed with ES from 2010 to 2016 in the SEER database were screened by SEER ^*^ STAT (version 8.3.5) software. 929 patients were selected as training cohort according to inclusion/exclusion criteria: (1) ES confirmed according to morphological code 60 of ICD-O-3/WHO 2008; (2) The patients who did not lose follow-up; (3) A clear description of tumor invasion; (4) Complete baseline information; (5) Clear treatment information; (6) Survival time is >0.

The information on sex, age, race, survival time, primary site, laterality, T stage, N stage, surgery, radiotherapy, chemotherapy, and distant metastasis were collected. External verification using data of 51 ES patients via clinical and pathological diagnosis from four medical institutions (the Second Affiliated Hospital of Jilin University, the Second Affiliated Hospital of Dalian Medical University, Liuzhou People's Hospital, and Xianyang Central Hospital). Three investigators were responsible for data acquisition and processing at each center. Two of them were in charge of extracting the data while the third person was responsible for further verification. Data was further checked by using Microsoft Excel (Microsoft Excel, 2013, Redmond, USA).

### Construction and Verification of the Nomogram

We compared the baseline information of 980 patients using the *T*-test and Chi-square test. Then we evaluated several potential factors to predict lymphatic metastasis in ES patients by univariate logistic regression analysis. Multivariate logistic regression was utilized to further analyze the independent factors associated with lymphatic metastasis in ES patients. From this, we constructed a nomogram, plotted the receiver operating characteristic (ROC) curve, and calculated the area under the curve (AUC) to evaluate the accuracy of nomogram. The proximity between the actual probability and the predicted probability was verified by the calibration plot. Decision curve analysis (DCA) was used to evaluate the clinical utility.

### Statistical Analysis

Quantitative data were calculated in the form of Mean ± standard deviation (SD), and qualitative data were expressed as counts and percentages. SPSS 26.0 software (SPSS Inc., Chicago, USA) was used for *T*-test, Chi-square test, Kaplan-Meier, univariate and multivariate logistic regression analysis. Nomogram, ROC curve, calibration plot, and DCA curve were prepared by R language (version 4.0.5). *P* < 0.05 was considered statistically significant.

## Results

### Baseline Information

A total of 980 patients were included, among which 929 patients were screened from the SEER database for a training cohort and the other 51 patients were from four clinical centers for validation. The results of the *T*-test and Chi-square test with baseline information of patients showed that no significant differences existed between the training group and the validation group in survival time, age, sex, primary site, laterality, M stage, surgery, chemotherapy and lung metastasis (Table 1, *P* > 0.05), however, differences were statistically significant in the race, radiotherapy and T stage statistically (Table 1, *P* < 0.05). Among the 980 patients, 139 of them had lymphatic metastasis, while 841 didn't have or the status of lymphatic metastasis could not be assessed. There were statistically significant differences between the two groups in survival time, race T stage, M stage, surgery and lung metastasis (Table 2, *P* < 0.05).

**Table 1:** Baseline data table of the training group and the validation group.

**Variable**	**Level**	**Overall** **(*N* = 980)**	**SEER** **(Training group, *N* = 929)**	**Multicenter data** **(Validation group, *N* = 51)**	***P*-value**
Lymph node metastases (%)	No	841 (85.82)	797 (85.79)	44 (86.27)	1.000
	Yes/unable.to.evaluate	139 (14.18)	132 (14.21)	7 (13.73)	
Race (%)	Black	39 (3.98)	39 (4.20)	0 (0.00)	<0.001
	White	815 (83.16)	815 (87.73)	0 (0.00)	
	Others	126 (12.86)	75 (8.07)	51 (100.00)	
Times [median (IQR^a^)]	NA	26.000 (11.000, 47.000)	26.000 (11.000, 47.000)	23.000 (12.500, 39.500)	0.851
Age [median (IQR)]	NA	17.000 (12.000, 27.000)	17.000 (12.000, 27.000)	17.000 (12.500, 30.500)	0.480
Sex (%)	Female	418 (42.65)	395 (42.52)	23 (45.10)	0.828
	Male	562 (57.35)	534 (57.48)	28 (54.90)	
Primary site (%)	Axis bone	431 (43.98)	404 (43.49)	27 (52.94)	0.394
	Limb bone	317 (32.35)	304 (32.72)	13 (25.49)	
	Other	232 (23.67)	221 (23.79)	11 (21.57)	
Laterality (%)	Left	374 (38.16)	353 (38.00)	21 (41.18)	0.895
	Unpaired sites	296 (30.20)	281 (30.25)	15 (29.41)	
	Right	310 (31.63)	295 (31.75)	15 (29.41)	
T stage^b^ (%)	T1	351 (35.82)	331 (35.63)	20 (39.22)	0.008
	T2	429 (43.78)	404 (43.49)	25 (49.02)	
	T3	39 (3.98)	34 (3.66)	5 (9.80)	
	TX	161 (16.43)	160 (17.22)	1 (1.96)	
M stage^c^ (%)	M0	662 (67.55)	632 (68.03)	30 (58.82)	0.225
	M1	318 (32.45)	297 (31.97)	21 (41.18)	
Surgery (%)	No	413 (42.14)	388 (41.77)	25 (49.02)	0.381
	Yes	567 (57.86)	541 (58.23)	26 (50.98)	
Radiotherapy (%)	No	757 (77.24)	728 (78.36)	29 (56.86)	<0.001
	Yes	223 (22.76)	201 (21.64)	22 (43.14)	
Chemotherapy (%)	No/Unknown	58 (5.92)	58 (6.24)	0 (0.00)	0.125
	Yes	922 (94.08)	871 (93.76)	51 (100.00)	
Lung metastases (%)	No	795 (81.12)	754 (81.16)	41 (80.39)	1.000
	Yes	185 (18.88)	175 (18.84)	10 (19.61)	

a *Inter-Quartile Range*.

b *Tumer stage*.

c *Metastasis stage*.

**Table 2:** Patient baseline table of lymphatic metastases.

**Variable**	**Level**	**Overall (*N* = 980)**	**No (*N* = 841)**	**Yes/unable.to.evaluate** **(*N* = 139)**	***P*-value**
Category (%)	Multicenter data (Validation group)	51 (5.2)	44 (5.2)	7 (5.0)	1.000
	SEER (Training group)	929 (94.8)	797 (94.8)	132 (95.0)	
Times [mean (SD^a^)]	NA	30.56 (22.65)	31.95 (22.72)	22.17 (20.32)	<0.001
Race (%)	Black	39 (4.0)	28 (3.3)	11 (7.9)	0.037
	White	815 (83.2)	705 (83.8)	110 (79.1)	
	Others	126 (12.9)	108 (12.8)	18 (12.9)	
Age [mean (SD)]	NA	22.39 (16.45)	22.26 (16.37)	23.19 (16.97)	0.533
Sex (%)	Female	418 (42.7)	368 (43.8)	50 (36.0)	0.104
	Male	562 (57.3)	473 (56.2)	89 (64.0)	
Primary.site (%)	Axis bone	431 (44.0)	365 (43.4)	66 (47.5)	0.493
	Limb bone	317 (32.3)	278 (33.1)	39 (28.1)	
	Others	232 (23.7)	198 (23.5)	34 (24.5)	
Laterality (%)	Left	374 (38.2)	325 (38.6)	49 (35.3)	0.375
	Unpaired sites	296 (30.2)	247 (29.4)	49 (35.3)	
	Right	310 (31.6)	269 (32.0)	41 (29.5)	
T stage (%)	T1	351 (35.8)	322 (38.3)	29 (20.9)	<0.001
	T2	429 (43.8)	362 (43.0)	67 (48.2)	
	T3	39 (4.0)	34 (4.0)	5 (3.6)	
	TX	161 (16.4)	123 (14.6)	38 (27.3)	
M stage (%)	M0	662 (67.6)	605 (71.9)	57 (41.0)	<0.001
	M1	318 (32.4)	236 (28.1)	82 (59.0)	
Surgery (%)	No	413 (42.1)	337 (40.1)	76 (54.7)	0.002
	Yes	567 (57.9)	504 (59.9)	63 (45.3)	
Radiotherapy (%)	No	757 (77.2)	647 (76.9)	110 (79.1)	0.642
	Yes	223 (22.8)	194 (23.1)	29 (20.9)	
Chemotherapy (%)	No/unknown	58 (5.9)	47 (5.6)	11 (7.9)	0.378
	Yes	922 (94.1)	794 (94.4)	128 (92.1)	
Lung.metastases (%)	No	795 (81.1)	713 (84.8)	82 (59.0)	<0.001
	Yes	185 (18.9)	128 (15.2)	57 (41.0)	

a *Standard deviation*.

*T stage and M stage are same in Table 1*.

### Univariate and Multivariate Logistic Regression Analysis

Univariate logistic regression analysis showed that survival time, race, T stage, M stage, surgery, and lung metastasis were significant factors for lymphatic metastasis in ES patients (Table 3). Further multivariate logistic regression analysis of the data indicated that T stage (Tx OR = 2.540, 95%CI = 1.433–4.503, *P* < 0.01), M stage (M1, OR = 2.061, 95%CI = 1.189–3.573, *P* < 0.05) and survival time (OR = 0.982, 95%CI = 0.972–0.992, *P* < 0.001) were important independent factors for lymphatic metastasis in ES patients (Table 3).

**Table 3:** Univariate and multivariate logistic regression analysis of risk factors for lymphtic metastases in patients with Ewing's sarcoma.

**Variables**	**Univariate OR** **(95% CI)**	***P*-value**	**Multivariate OR** **(95% CI)**	***P*-value**
Age (years)	1.005 (0.994–1.016)	0.371	/	/
Survival.time (month)	0.977 (0.967–0.986)	<0.001	0.982 (0.972–0.992)	<0.001
**Race**
White	Ref	Ref	Ref	Ref
Black	2.518 (1.218–5.203)	<0.050	1.958 (0.889–4.310)	0.095
Other	1.102 (0.563–2.154)	0.777	1.100 (0.544–2.222)	0.791
**Sex**
Male	Ref	Ref	Ref	Ref
Female	0.741 (0.506–1.085)	0.123	/	/
**Primary site**
Limb bones	Ref	Ref	Ref	Ref
Axis of a bone	1.308 (0.845–2.026)	0.229	/	/
Other	1.267 (0.764–2.099)	0.359	/	/
**Laterality**
Left	Ref	Ref	Ref	Ref
Right	0.968 (0.615–1.524)	0.888	/	/
Other	1.121 (0.780–1.883)	0.393	/	/
**T stage**
T1	Ref	Ref	Ref	Ref
T2	2.167 (1.338–3.510)	<0.010	1.630 (0.985–2.699)	0.057
T3	2.023 (0.722–5.666)	0.180	0.877 (0.297–2.595)	0.813
TX	3.645 (2.127–6.278)	<0.001	2.540 (1.433–4.503)	<0.010
**M stage**
M0	Ref	Ref	Ref	Ref
M1	3.672 (2.513–5.365)	<0.001	2.061 (1.189–3.573)	<0.050
**Surgery**
No	Ref	Ref	Ref	Ref
Yes	0.547 (0.378–0.793)	<0.01	1.024 (0.663–1.581)	0.916
**Radiotherapy**
No	Ref	Ref	Ref	Ref
Yes	0.872 (0.550–1.382)	0.559	/	/
**Chemotherapy**
No	Ref	Ref	Ref	Ref
Yes	0.689 (0.348–1.366)	0.286	/	/
**Lung metastases**
No	Ref	Ref	Ref	Ref
Yes	3.868 (2.600–5.754)	<0.001	1.724 (0.974–3.051)	0.061

*Ref, reference*.

*T stage and M stage are same in Table 1*.

**Figure 1:**
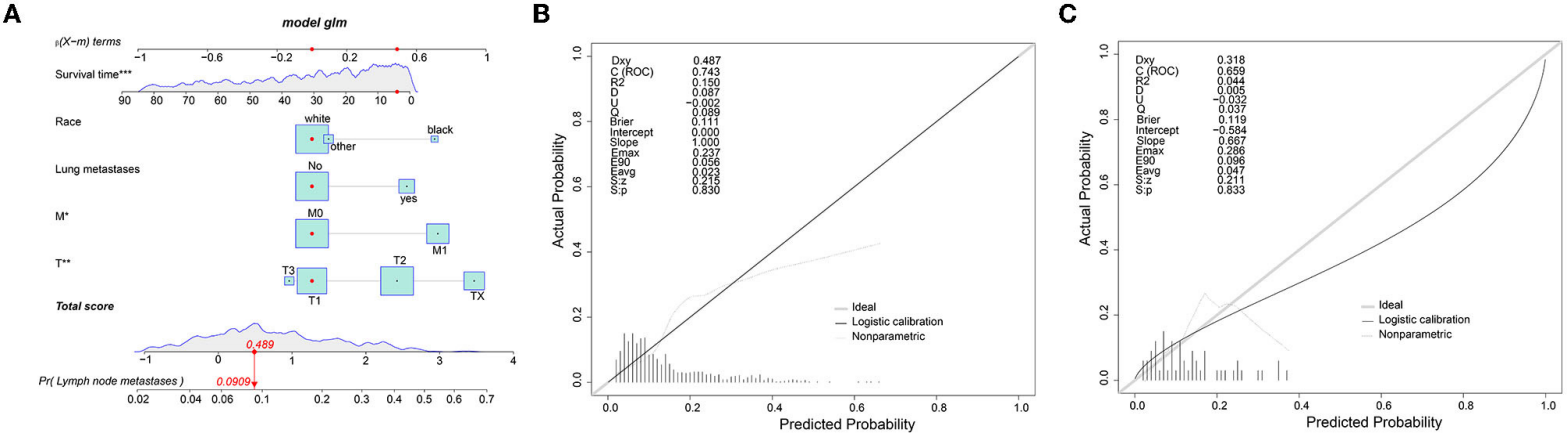
**(A)** The nomogram for the risk of lymphatic metastasis for ES patients. **(B)** The calibration plots of the training cohort. **(C)** The calibration plots of the validation cohort.

**Figure 2:**
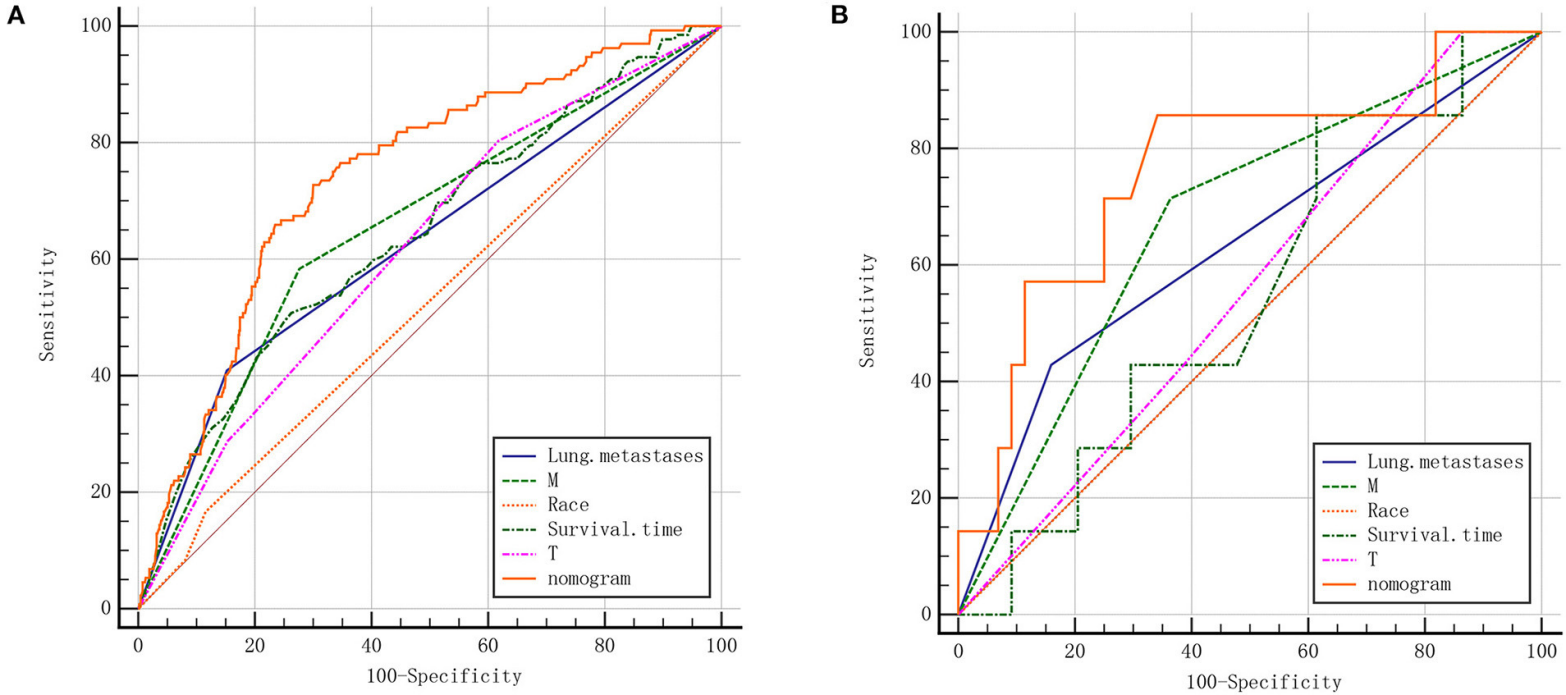
**(A)** ROC of the nomogram for the training cohort. **(B)** ROC of the nomogram for the validation cohort.

To predict the risk of lymphatic metastasis in patients with ES, we constructed a nomogram based on the results of logistic regression (Figure 1A). Meanwhile, we designed an online calculator (https://drliwenle.shinyapps.io/LMES/) in order to assess the risk of lymphatic metastases. We found that Tx had the greatest impact on lymphatic metastasis while surgery had the least impact (Figure 1A). The AUC of internal validation was 0.743 (95% CI: 0.714–0.771), while that of external validation was 0.763 (95%CI: 0.623–0.871) (Table 4), indicating that the accuracy of the assessment of lung metastasis in ES patients was relatively high in the nomogram and the model we constructed was more accurate than the single-factor prediction (Figures 2A,B). The calibration plot showed good consistency and the prediction was close to the actual situation (Figures 1B,C).

**Table 4:** AUC of training group and validation group.

	**SEER data**			**Multicenter data**		
	**(Training group)**			**(Validation group)**		
**Variable**	**AUC**	**SE**	**95% CI**	**AUC**	**SE**	**95% CI**
Lung.metastases	0.629	0.022	0.597–0.660	0.635	0.105	0.488–0.765
M stage	0.654	0.023	0.622–0.684	0.675	0.099	0.530–0.800
Race	0.524	0.017	0.491–0.556	0.500	0.000	0.357–0.643
Survival.time	0.645	0.027	0.613–0.676	0.549	0.113	0.403–0.688
T stage	0.620	0.024	0.588–0.651	0.560	0.098	0.414–0.699
Nomogram	0.743	0.023	0.714–0.771	0.763	0.111	0.623–0.871

*AUC, area under curve; SE, standard error; CI, confidence interval*.

*T stage and M stage are same in Table 1*.

### Clinical Application of the Nomogram

**Figure 3:**
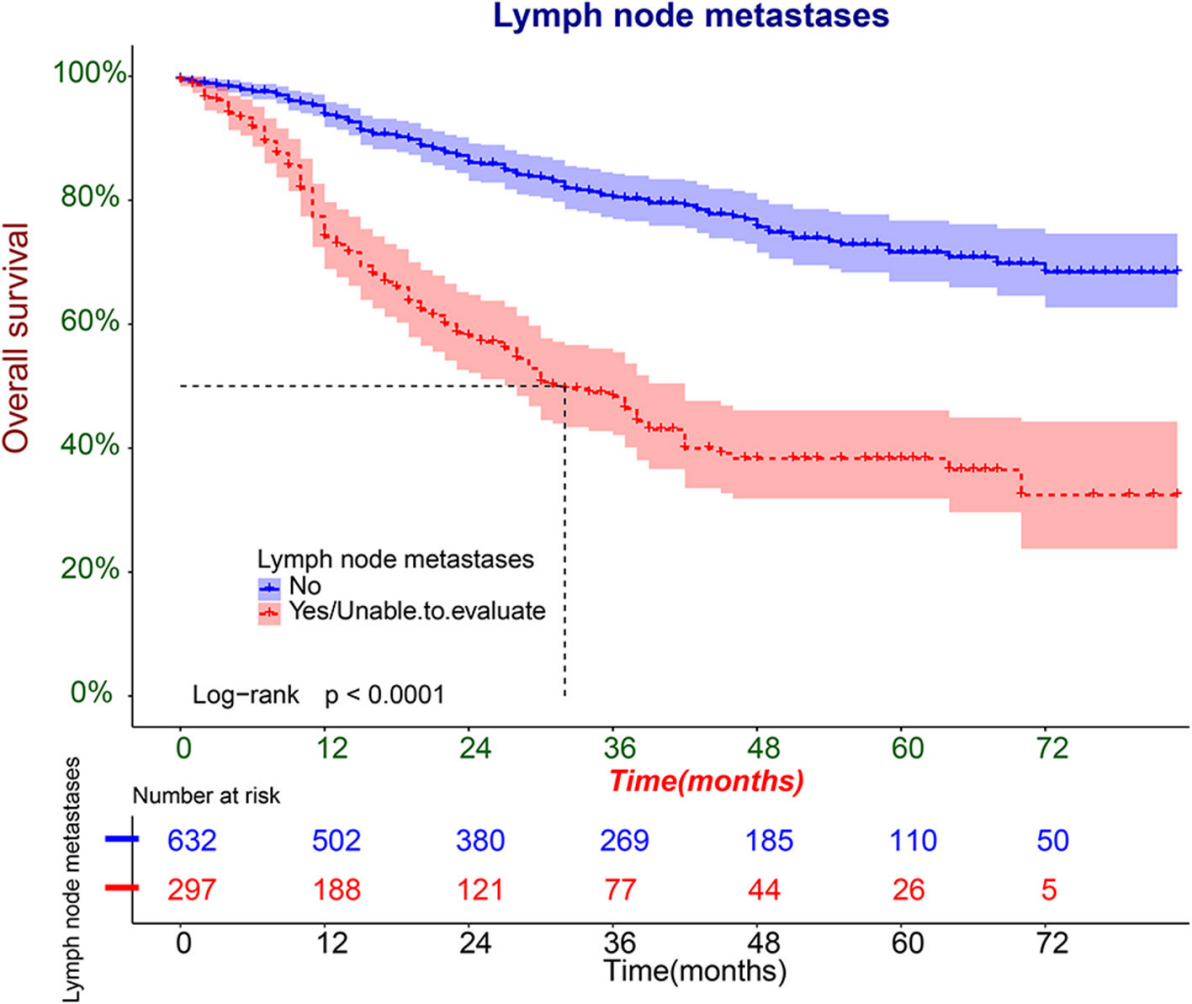
The Kaplan-Meier overall survival (OS) analysis of lymphatic metastasis in patients with ES.

**Figure 4:**
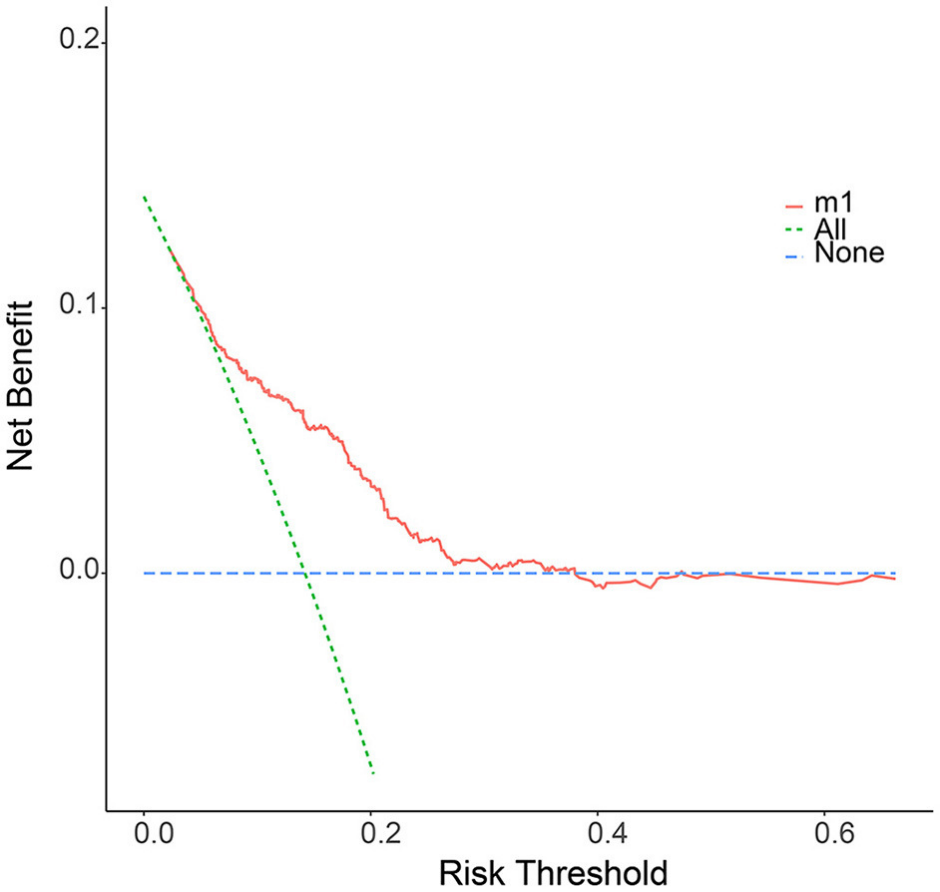
The decision curve (DCA) of nomogram for the risk of lymphatic metastasis. The red curve displays the number of people classified as positive by nomogram for each threshold probability. The green curve shows the number of true positives under each threshold probability.

We further plotted Kaplan-Meier overall survival (OS) curves for patients with and without lymphatic metastasis (Figure 3). Compared to the survival time of patients with lymphatic metastasis, survival time in patients with lymphatic metastasis or unclear situation (*P* < 0.0001) was significantly lower. At the same time, DCA plots were drawn to evaluate the clinical utility of the model, and we observed that the model had good clinical utility in predicting lymphatic metastasis in ES patients (Figure 4).

## Discussion

Ewing's sarcoma (ES), first reported by James Ewing in 1921, is an aggressive bone and soft-tissue tumor (5, 9, 22, 23). Thanks to a variety of treatment measures have been adopted in ES, the prognosis of patients with localized forms of the tumor has been significantly improved. However, the 5-year survival rate for metastasis in patients is still lower than 30% (5). Therefore, it is essential to seek more effective treatment methods to prolong the survival time of patients and improve the prognosis of patients. Some scholars pointed out that the clinical management and prognosis of ES could benefit from the biological markers, nevertheless, there was no biomarker that could really predict the prognosis of patients (24). Advancements in artificial intelligence (AI) and machine learning approach can also help clinicians with the timely diagnosis and more efficient treatment of patients in the healthcare sector (25–27). Nomogram was much more convenient to obtain the predictors. Hence, it was considered a big advantage over traditional staging, which had been proposed as an alternative and even a new standard to guide the treatment of cancer patients (17, 28, 29). We screened the clinical information of 929 patients with ES using a SEER database and found that survival time, race, T stage, M stage, surgery, and lung metastasis were significant factors that affected lymphatic metastasis in ES patients. In view of the prognostic factors above, the integration of the nomogram could be intuitively used in the clinical evaluation of lymphatic metastasis in patients with ES. After the model was established, we collected the data of 51 patients from four clinical centers and conducted external validation to confirm the reliability of the model, thus enhancing the value of the model in clinical application. Besides, we established a web calculator for the prediction and evaluation of lymphatic metastasis in ES patients.

Logistic regression analysis indicated that T stage, M stage and survival time were independent factors for lymphatic metastasis in ES patients. Several consistent prognostic factors were identified in North America and Europe. Multiple studies showed that in addition to stage, poor prognostic factors included tumor size and metastasis at diagnosis (30–32), which were basically consistent with our findings.

Tumor size was considered to be an important prognostic factor. Large initial tumor size has been repeatedly identified as negatively affecting survival in ES patients (7). The correlation between tumor size and metastasis remains to be further studied. We hypothesized that the larger tumor size may have more potential invasion and metastasis, and the existence of abundant lymphatic tissue also promotes the occurrence of lymphatic metastasis.

The current study also showed that most patients began with micro-metastases (33). Furthermore, ES had a wide range of metastasis sites, including lung, bone, lymph nodes, liver and brain (1). Bone and lung metastases and the development of extensive tumors were linked with poor prognosis. Patients were most likely to develop lung metastases than bone metastases, and patients with isolated lung metastases had a better prognosis than those with extra-pulmonary metastases. Patients with unilateral lung involvement appeared better than those with bilateral lung (34). Our study also revealed that M stage was a key predictor. In this study, about 59% of patients with lymphatic metastasis developed the state of M1, and the OR of lymphatic metastasis in M1 patients was 2.061. Moreover, patients with lymphatic metastasis have a higher tendency to develop lung metastasis, which is consistent with the results of logistics regression analysis. For the risk of metastasis of the confirmed patients, systemic staging can be performed with CT and MRI for primary site, and ^18^F-fluorodeoxyglucose(FDG)PET-CT scan.

Based on previous studies, ES was more commonly found in men than in women (sex ratio of 3:2), and the prevalence was over seven times higher in whites than in blacks (1, 5, 35). In addition, Duchman et al. found that blacks had an increased frequency of metastases when diagnosed with ES compared to patients of other races (36). Our results showed that the incidence of lymphatic metastasis was more than 2 times higher in black patients than in white patients, and 1.3 times higher in men than in women.

Although this model provided theoretical support for clinical diagnosis and treatment, some shortcomings remained in our study: (1) first of all, although we established the lymphatic metastasis in patients with clinical prediction model which was verified by external cohort, external validation group of small number of cases might be a problem such as selective bias; (2) due to incomplete data entry in SEER database, we were unable to analyze all factors that might be associated with lymphatic metastasis. For instance, it was lack of some laboratory indicators, such as serum lactate dehydrogenase (LDH) levels. Based on this, we could avoid bias by further increasing the number of cases in the external cohort. In addition, we could use the case data of Asian people to establish a more suitable prediction model for Asian people so that we can make a comparison of this model.

## Conclusion

Our study established the associated nomogram by using data from the SEER database and comprehensively evaluated predictors of lymphatic metastasis in ES patients via external validation. The prediction of the risk of lymphatic metastasis could guide clinicians to carry out individualized and precise treatment for patients, and help to formulate follow-up treatment measures and plans for patients.

## Data Availability Statement

The original contributions presented in the study are included in the article/Supplementary Material, further inquiries can be directed to the corresponding authors.

## Ethics Statement

All participants signed written informed consents following the recommendations of the Institutional Review Board of the Xianyang Central Hospital. The ethical approval certificate is 20210022.

## Author Contributions

CY and ZL designed the study. WeL and ZH performed the study and analyzed the data. WeL and CX wrote the manuscript. SD, HW, QL, and Z-RT provided the expert consultations and clinical suggestions. WaL participated in its design and coordination. BW helped to draft the manuscript. All authors reviewed the final version of the manuscript.

## Conflict of Interest

The authors declare that the research was conducted in the absence of any commercial or financial relationships that could be construed as a potential conflict of interest.

## Publisher's Note

All claims expressed in this article are solely those of the authors and do not necessarily represent those of their affiliated organizations, or those of the publisher, the editors and the reviewers. Any product that may be evaluated in this article, or claim that may be made by its manufacturer, is not guaranteed or endorsed by the publisher.
